# IgG4-related disease involving the penis: a rare case report

**DOI:** 10.3389/fimmu.2026.1757885

**Published:** 2026-03-17

**Authors:** Junjie Chen, Jinlu Sun, Qichao Chen, Wei Zhang, Qiuyuan Xia, Haowei He

**Affiliations:** 1 Department of Urology, Jinling Clinical Medical College, Nanjing Medical University, Nanjing, Jiangsu, China; 2 Department of Urology, Jinling Hospital, Affiliated Hospital of Medical School, Nanjing University, Nanjing, Jiangsu, China; 3 Department of Pathology, Jinling Hospital, Affiliated Hospital of Medical School, Nanjing University, Nanjing, Jiangsu, China

**Keywords:** case report, glucocorticoids, IgG4-related disease, inflammatory mass, penis

## Abstract

Immunoglobulin G4-related disease (IgG4-RD) is a chronic, systemic fibroinflammatory autoimmune disorder characterized by multi-organ involvement, elevated serum IgG4 levels, and dense infiltration of IgG4^+^ plasma cells. The disease can affect nearly any organ, and its clinical spectrum has continued to expand in recent years; involvement of the genitourinary system has been increasingly recognized, although direct involvement of the male external genitalia is extremely rare. We report a case of a 29-year-old man with IgG4-RD who initially presented with an unexplained penile mass. Clinical evaluation and laboratory testing revealed right inguinal lymphadenopathy along with elevated serum IgG4 and C-reactive protein (CRP) levels. Histopathological examination demonstrated fibrosis and an increased number of IgG4^+^ plasma cells, confirming the diagnosis of IgG4-RD. The patient received prednisone at 30 mg/day for four consecutive weeks, which resulted in a marked reduction of the lesion and improvement in symptoms, followed by a taper to a maintenance dose of 6 mg/day. During follow-up, no recurrence of symptoms has been observed. This case indicates that IgG4-RD should be considered in patients presenting with unexplained penile masses, and early recognition with multidisciplinary collaboration among urology, pathology, and rheumatology is essential to prevent unnecessary surgical procedures.

## Introduction

Immunoglobulin G4-related disease (IgG4-RD), first described in 2003, is an immune-mediated fibroinflammatory disorder that predominantly affects middle-aged and older men (1, 2). It is characterized by tumefactive lesions, elevated serum IgG4 levels, and dense infiltration of IgG4^+^ plasma cells (3). The pancreas, salivary glands, retroperitoneum, and kidneys are most commonly involved. Although genitourinary manifestations such as retroperitoneal fibrosis, ureteritis, prostatitis, and epididymitis have been described (4, 5), direct involvement of the penis, has not been previously reported. This case aims to broaden the clinical spectrum of IgG4-RD and enhance clinicians’ ability to recognize this immune-mediated condition.

## Case presentation

**Figure 1:**
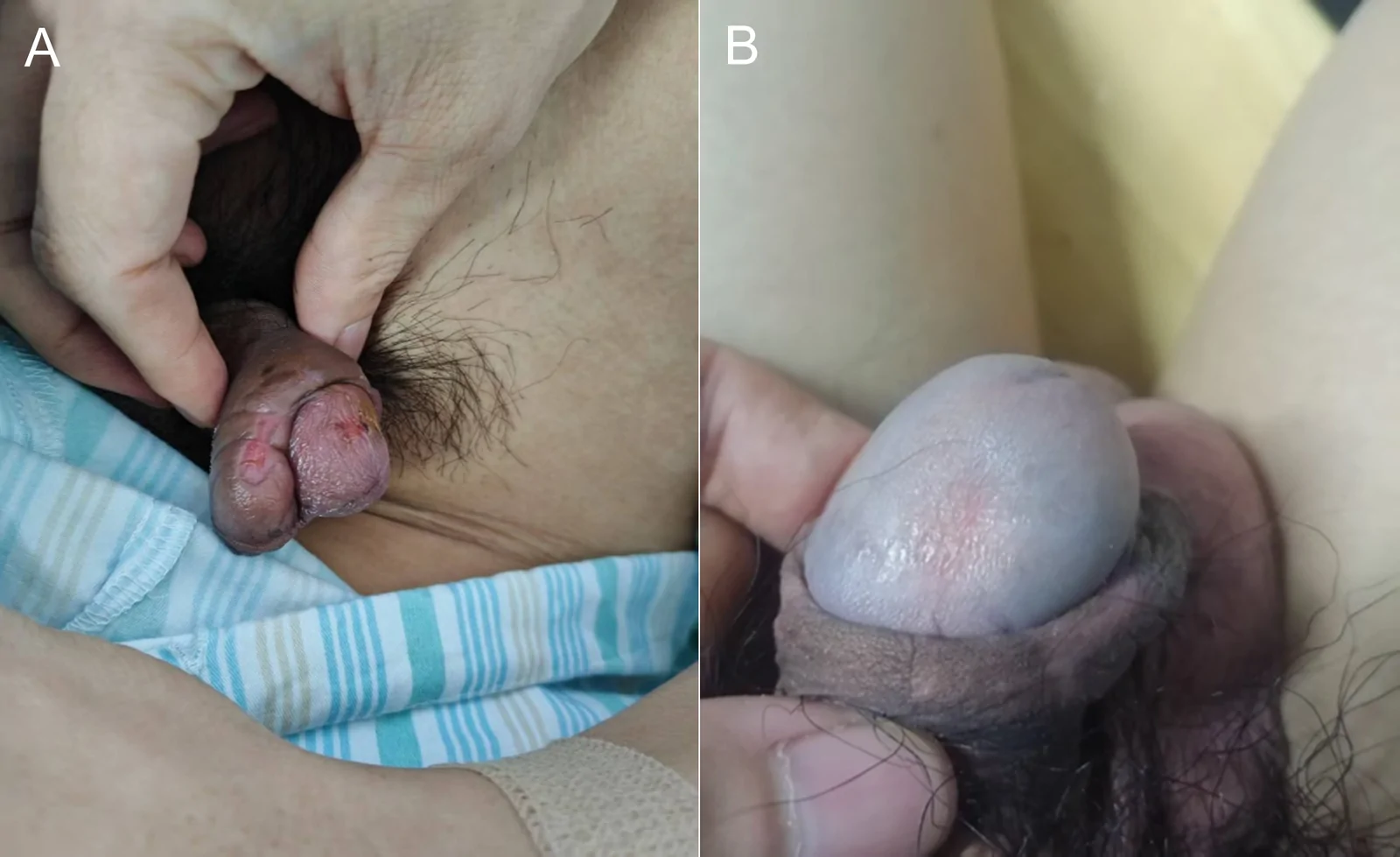
Lesion before and after treatment. **(A)** A novel mass is visible on the penis, showing necrosis and erosion of the foreskin and glans (pre-treatment lesion). **(B)** Follow-up clinical photograph demonstrates significant reduction in the size of the penile mass and complete resolution of the cutaneous and mucosal lesions (post-treatment lesion).

A 29-year-old man presented to our urology department on October 12, 2024, with a one-month history of a penile mass. The lesion appeared on the dorsal aspect of the penis near the coronal sulcus without an obvious trigger. It was initially painless but progressively enlarged to approximately 3 cm × 3 cm, with a firm consistency and a relatively smooth surface. At onset, the patient reported no urinary symptoms, sexual dysfunction, or penile discharge. Starting October 13, he developed fever with a maximum temperature of 38.6 °C, accompanied by a tender mass in the right inguinal region. Physical examination revealed enlargement of the right inguinal lymph nodes (approximately 2.5 cm × 1.8 cm) and patchy necrotic changes with mild exudation on the ipsilateral glans and prepuce. (**Figure 1A**).

Physical examination was otherwise normal. Laboratory tests showed leukocytosis with a white blood cell count of 10.7 × 10^9^/L (reference range: 3.5–9.5 × 10^9^/L), decreased hemoglobin at 119 g/L (reference range: 130–175 g/L), elevated C-reactive protein at 80.61 mg/L (reference range: 0–8 mg/L), and neutrophilia with a neutrophil count of 7.9 × 10^9^/L (reference range: 1.8–6.3 × 10^9^/L). Chest computed tomography (CT) revealed minor inflammatory changes in the lower lobes of both lungs. Enhanced abdominal CT demonstrated a mass on the right anterior aspect of the penis, appearing as a low-density lesion with unclear margins, along with multiple enlarged lymph nodes adjacent to the right iliac vessels and in the inguinal region; small lymph nodes were also observed at the hepatic hilum and in the retroperitoneum. Positron emission tomography–computed tomography (PET-CT) showed increased 18F-fluorodeoxyglucose (FDG) uptake in the penile lesion (SUVmax 5.8), indicating high metabolic activity. A malignant tumor, such as sarcoma or lymphoma, was initially suspected.

To establish a definitive diagnosis, a biopsy of the penile lesion was performed. Histopathological examination revealed squamous epithelial hyperplasia and diffuse infiltration of lymphocytes and plasma cells within the stroma, accompanied by a small number of eosinophils and focal fibrosis. Immunohistochemical (IHC) staining showed CD20 (focal positive), CD3 (1+), CD138 (3+), CD38 (3+), and both κ and λ light chains were positive, indicating a polyclonal pattern. Epstein-Barr virus-encoded RNA (EBER) *in situ* hybridization was negative, and the Ki-67 proliferation index was approximately 10%, with no evidence of atypical lymphocytes or malignancy.

Further serological testing revealed elevated IgG at 21.47 g/L (reference range: 6.8–14.45 g/L), complement component 3 (C3) at 2.54 g/L (reference range: 0.8–1.6 g/L), and complement component 4 (C4) at 0.50 g/L (reference range: 0.09–0.36 g/L). Serum IgG4 reached 4635 μg/mL (normal value < 1350 μg/mL), representing a marked elevation.

Comprehensive autoimmune serologies were subsequently evaluated to exclude systemic autoimmune diseases, including antinuclear antibody (ANA), anti–double-stranded DNA antibody (anti-dsDNA), anti–Sjögren’s-syndrome–related antigen A antibody (anti-SSA/Ro), anti–Sjögren’s-syndrome–related antigen B antibody (anti-SSB/La), anti-Smith antibody (anti-Sm), and anti–RNP/Sm-related protein A antibody (A-RPA), all of which were negative. Antineutrophil cytoplasmic antibodies (ANCA), including cytoplasmic (cANCA), perinuclear (pANCA), and atypical patterns, as well as anti-myeloperoxidase antibody (MPO-ANCA), anti-proteinase 3 antibody (PR3-ANCA), and anti–glomerular basement membrane antibody (A-GBM), were also negative. The patient had no history of inflammatory bowel disease, asthma, or other atopic/allergic conditions.

**Figure 2:**
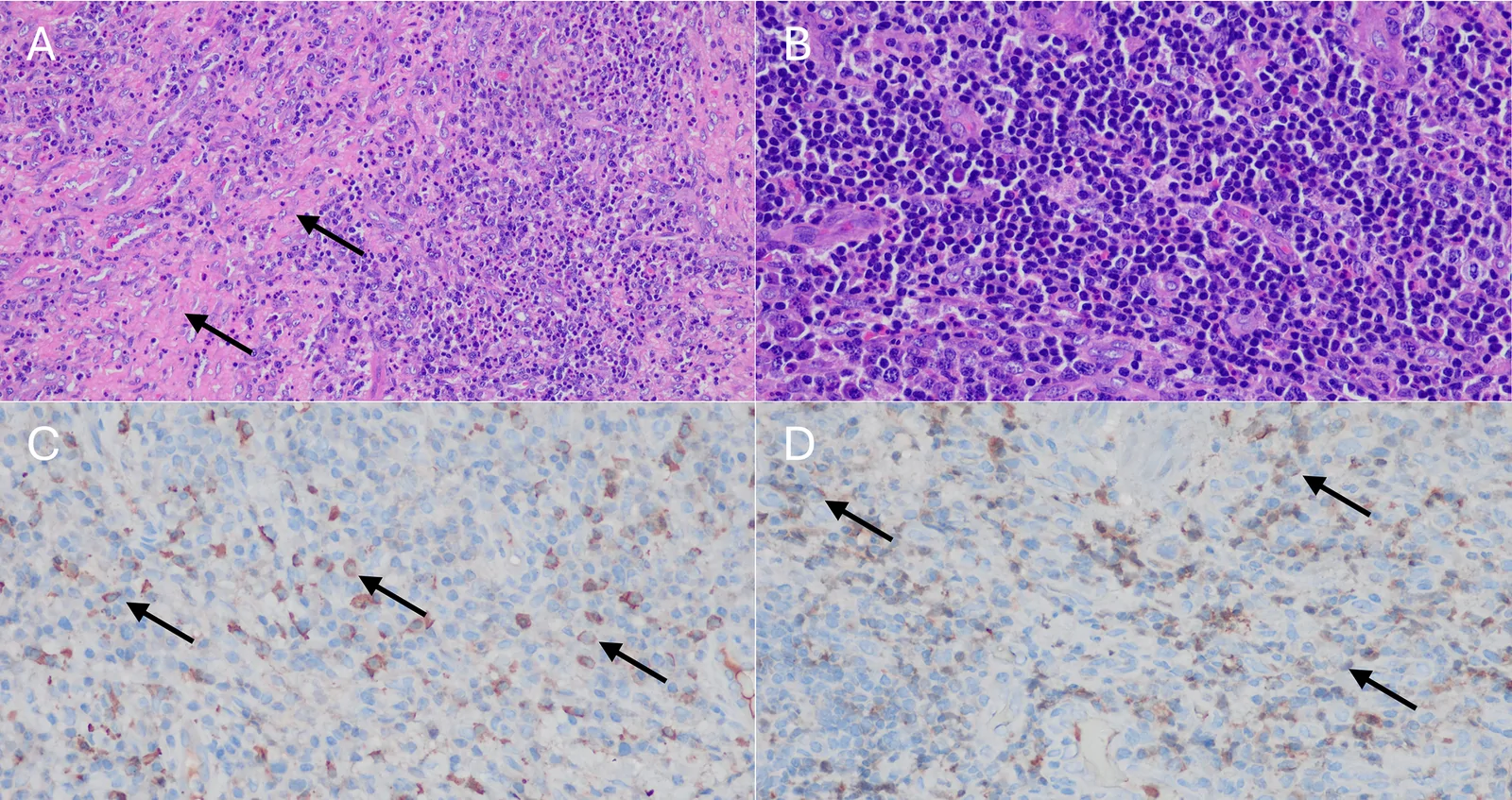
Histopathological and immunohistochemical findings of the penile lesion. **(A)** H&E staining (×200) showing focal fibrosis (arrows), accompanied by dense lymphoplasmacytic infiltration. **(B)** H&E staining (×400) demonstrating dense lymphoplasmacytic infiltration composed predominantly of lymphocytes and plasma cells. **(C)** Immunohistochemical staining for IgG4 (×400) showing numerous IgG4^+^ plasma cells (arrows). **(D)** Immunohistochemical staining for IgG (×400) demonstrating abundant IgG^+^ plasma cells (arrows).

Histopathological examination of the lesion revealed dense lymphoplasmacytic infiltration accompanied by focal fibrosis (**Figures 2A, B**). Typical obliterative phlebitis and storiform fibrosis were not identified. No significant eosinophil infiltration or non-obliterative phlebitis was observed. Additional immunohistochemical (IHC) staining for IgG and IgG4 was performed. In areas with the highest density of plasma cell infiltration (hotspots), three representative high-power fields (HPFs, ×400) were selected for evaluation. These fields demonstrated a mean of more than 50 IgG4^+^ plasma cells per HPF, and the mean IgG4^+^/IgG^+^ plasma cell ratio exceeded 70% (**Figures 2C, D**). According to the 2019 American College of Rheumatology/European League Against Rheumatism (ACR/EULAR) classification criteria (6), the patient scored 34 points, exceeding the diagnostic threshold of 20 points and supporting the diagnosis of IgG4-RD, as shown in **Table 1**.

**Table 1:** The patient’s 2019 ACR/EULAR IgG4-related disease classification score.

Items	Details	Weight
Histopathology	Immunostaining (IgG4^+^/IgG^+^ ratio≥71% & IgG4^+^ cells/hpf ≥51)	16
Serum IgG4 concentration	2–5× upper limit of normal	6
Bilateral lacrimal, parotid, sublingual and submandibular glands	No set of glands involved	0
Chest	Peribronchovascular and septal thickening	4
Pancreas and biliary tree	Not checked or none of the items listed is present	0
Kidney	Renal pelvis thickening/soft tissue	8
Retroperitoneum	Not checked or none of the items listed is present	0
Total inclusion points		34

A cumulative score of 20 points is required for the diagnosis. IgG4, Immunoglobulin G4; IgG, Immunoglobulin G; hpf, high-power field.

Initial treatment with intravenous hydrocortisone 30 mg/day led to marked shrinkage of the penile mass and regression of the right inguinal lymphadenopathy, after which the patient was transitioned to oral prednisone. On December 6, 2024, he developed acute left hydronephrosis with abdominal colic due to thickening of the renal pelvis and ureter, and underwent left ureteral double-J stent placement to relieve urinary obstruction. Considering high disease activity and evidence of multi-organ involvement, the rheumatology team was consulted on December 16, 2024, and the treatment regimen was adjusted to methotrexate 10 mg orally once weekly, hydroxychloroquine sulfate 0.2 g twice daily, and folic acid 5 mg daily to prevent myelosuppression. The patient was discharged on December 20, 2024, and continued outpatient follow-up.

We implemented a strategy of steroid-induced remission followed by a tapering regimen for maintenance. Prednisone was maintained at 30 mg/day for four weeks, then tapered as follows: reduced by 4 mg every ten days until reaching 20 mg/day, then by 2 mg every ten days to 12 mg/day, and finally by 1 mg every ten days to a maintenance dose of 6 mg/day, with further adjustments as needed. Methotrexate and hydroxychloroquine were continued for disease control. Currently, the patient is maintained on prednisone 6 mg/day combined with immunosuppressants, with stable disease and no new symptoms.

Subsequently, at the January 2025 follow-up, serum IgG4 had risen to 7089 μg/mL. He therefore received short-term pulse methylprednisolone (40 mg/day intravenously) combined with mycophenolate mofetil at a dose of 0.75 g twice daily (total 1.5 g/day), resulting in a decrease of IgG4 to 6397 μg/mL. The ureteral stent was eventually removed on March 5, 2025.

**Figure 3:**
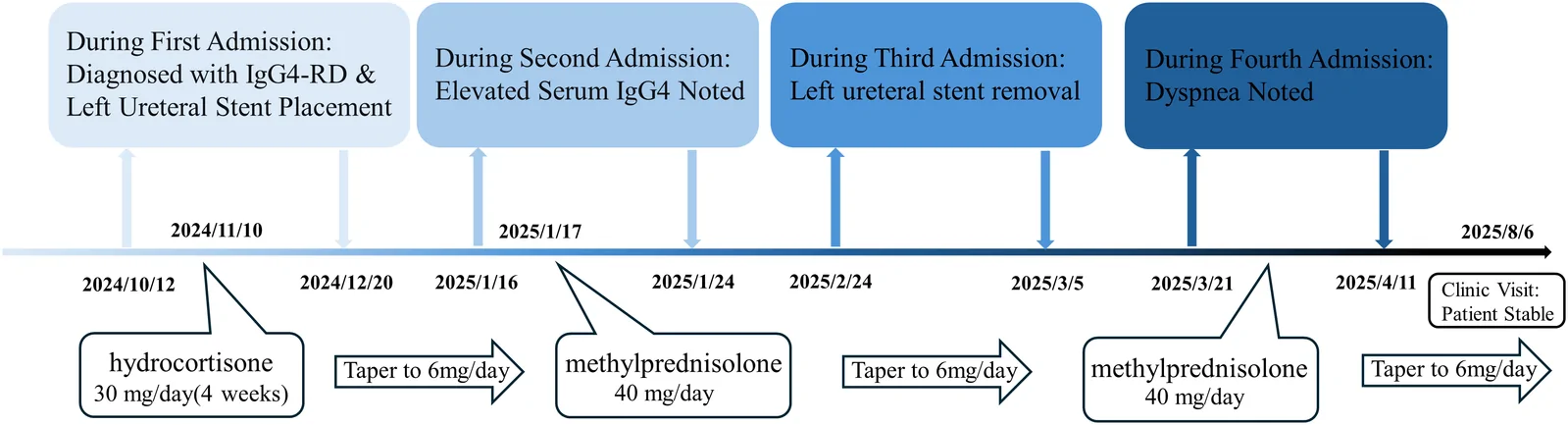
The timeline of diagnosis and treatment.

On March 21, 2025, he was admitted for dyspnea. Chest CT showed multifocal pulmonary lesions, and Mycoplasma pneumoniae IgM was positive, suggesting infection possibly related to the use of corticosteroids. He improved after anti-infective therapy and adjustment of immunosuppressive treatment and was discharged on April 11, 2025. During follow-up, serum IgG4 remained elevated, but there was no clinical or radiological relapse. We conducted continuous follow-up, and as of now, the patient remains in good condition. The timeline of diagnosis and treatment is summarized in **Figure 3**.

## Discussion

In the genitourinary system, IgG4-RD most commonly presents as retroperitoneal fibrosis, ureteral wall thickening, or tubulointerstitial nephritis (7). Patients with involvement of different organs often exhibit variable and non-specific symptoms, which creates substantial diagnostic challenges and may even lead to misdiagnosis or inappropriate treatment. This case highlights an unusual manifestation involving the external genitalia, resulting in significant diagnostic difficulty. The lesion appeared as a rapidly growing, metabolically active soft-tissue mass, closely resembling a malignant tumor. Moreover, because this patient lacked the characteristic systemic features of IgG4-RD—such as involvement of the pancreas or salivary glands—and presented with non-specific clinical findings, a broad range of differential diagnoses had to be considered.

Differential diagnoses included a spectrum of conditions that can present with penile masses, such as penile squamous cell carcinoma, lymphoma, and infectious granulomas (e.g., syphilis, rapid plasma reagin [RPR], and T-SPOT.TB [T-cell spot test for tuberculosis]). Penile squamous cell carcinoma was considered because the lesion exhibited progressive enlargement and a firm consistency. However, histopathological examination showed no evidence of epithelial dysplasia, keratinization, or ulcerative changes that would support a malignant epithelial process. Lymphoma was also contemplated given the presence of regional lymphadenopathy and increased metabolic activity on positron emission tomography–computed tomography, but immunohistochemical evaluation demonstrated polyclonal κ and λ light-chain expression rather than a monoclonal pattern, effectively ruling out lymphoid malignancy. In addition, infectious etiologies were taken into account, as granulomatous processes may present with mass-like lesions. Nonetheless, serological testing for syphilis was negative, and no caseating granulomas or other features suggestive of tuberculosis or bacterial infection were observed on histopathology. The low Ki-67 proliferation index (approximately 10%) further supported a benign inflammatory process rather than a neoplastic or infectious one. Systemic autoimmune diseases were also considered in the differential diagnosis, and comprehensive immunological testing was negative. With no relevant prior medical history, these disorders were effectively excluded.

Notably, the patient experienced a transient fever at disease onset, with a maximum temperature of 38.6 °C, which resolved completely following appropriate antimicrobial and symptomatic treatment. Laboratory and imaging findings supported that the fever was secondary to a necrotic infection rather than intrinsic disease activity. Clinically, fever only carries exclusionary significance when no alternative explanation, such as infection, is identified (6). Therefore, the presence of infection in this case does not undermine the subsequent diagnostic evaluation. As prominent fever is not typically a defining feature of IgG4-RD (8), careful differentiation between infectious and inflammatory causes was essential to ensure diagnostic accuracy.

Diagnostic approaches for IgG4-RD have continued to shift over recent years, and both the 2020 Revised Comprehensive Diagnostic criteria (9) and the 2019 ACR/EULAR classification criteria (6) place similar weight on serology and histopathology. Serum IgG4 is routinely measured, yet its performance varies among studies. One cross-sectional cohort reported elevated values in 78.7% of confirmed cases (10), whereas others have described sensitivities approaching 90% with specificities around 60% (11). These differences likely arise from variation in disease distribution, study populations, and testing methods rather than instability of the marker itself. Markedly increased concentrations (>5000 mg/L) are far more informative and can reach a specificity close to 90% (12), which was relevant in our case. Even so, serum elevation alone is insufficient; a prospective UK cohort showed that only 22.4% of individuals with raised IgG4 ultimately met diagnostic criteria (13).

Because of these limitations, histopathological evaluation remains essential in differentiating IgG4-RD from other inflammatory or fibrotic conditions. The established major histopathological features include dense lymphoplasmacytic infiltrate, storiform fibrosis, and obliterative phlebitis (9, 14). However, these features may not be present in all organs or in every case (15), particularly at uncommon sites of involvement. The number of IgG4^+^ plasma cells may also vary depending on tissue type and biopsy depth, and IgG4-rich infiltrates can be observed in other inflammatory conditions (16). Therefore, the IgG4^+^/IgG^+^ plasma cell ratio serves as an important supportive parameter, with proportions above 40% generally considered supportive of IgG4-RD. In this case, the ratio exceeded 70%, providing strong pathological support when interpreted alongside the clinical and serological findings.

Histologically, dense lymphoplasmacytic infiltration and focal fibrosis were observed, whereas typical storiform fibrosis and obliterative phlebitis were not identified. Despite the absence of some classical histological features, this case demonstrates important supportive findings, including abundant IgG4^+^ plasma cell infiltration on high-power examination, markedly elevated serum IgG4 levels (4635 μg/mL), additional organ involvement with thickening of the renal pelvis and ureter resulting in hydronephrosis, and a favorable response to glucocorticoid therapy. According to the 2012 international consensus statement on IgG4-RD pathology (14), these findings are most consistent with “probable histological features of IgG4-related disease”.

The pathogenesis of penile involvement in IgG4-RD remains speculative, largely because such presentations are extremely rare and mechanistic data are limited. The penis contains abundant dense connective tissues—such as the tunica albuginea, corporal septae, and other fibrocollagenous structures—that may provide a permissive stromal substrate for IgG4-mediated fibroinflammatory reactions. Immunologically, IgG4-RD is characterized by activation of T follicular helper 2 (Tfh2) cells, clonal expansion of B cells, and differentiation of plasma cells toward the IgG4 subclass (17), all of which contribute to chronic inflammation and progressive tissue remodeling. In genetically susceptible individuals, we speculate that repeated local microtrauma, chronic irritation, or other forms of persistent antigenic stimulation could alter or expose basement membrane and collagen components within penile tissues, thereby initiating an abnormal immune response. This process may drive the recruitment and accumulation of IgG4^+^ plasma cells, promote cytokine-mediated fibrosis, and ultimately lead to the localized fibroinflammatory mass observed in this case.

Glucocorticoids are the first-line treatment for IgG4-RD, and most patients respond well to steroid therapy, with significant reductions in serum IgG4 levels. However, even in patients who achieve clinical remission, serum IgG4 levels may not return to normal (18). This may be due to the persistence of long-lived plasma cells continuously producing IgG4 (19). Importantly, serum IgG4 alone does not reliably reflect disease activity and should not be used as the sole basis for treatment escalation (20). In this case, markedly elevated serum IgG4 partially influenced the decision to intensify immunosuppressive therapy, but reliance on a single laboratory marker has inherent limitations. Therefore, treatment decisions and adjustments should be based on comprehensive assessment, integrating clinical manifestations, laboratory findings, and imaging evidence. Although rituximab has shown high efficacy in refractory IgG4-RD (21), our patient responded well to conventional combination therapy (**Figure 1B**), indicating that early multimodal intervention may reduce reliance on biologic agents in certain cases.

In summary, this case highlights that IgG4-RD should be considered in any unexplained penile mass with inflammatory histology. Multidisciplinary collaboration among urology, pathology, and rheumatology is crucial to avoid unnecessary surgery and enable timely immunosuppressive therapy. Furthermore, this case provides a clinical reflection: adjustments to immunosuppressive therapy should be based on a comprehensive and coherent body of clinical evidence, rather than guided solely by the elevation of a single biomarker, allowing for a more rational and balanced treatment approach in complex presentations of IgG4-RD.

## Data Availability

The raw data supporting the conclusions of this article will be made available by the authors, without undue reservation.
